# Role of WRKY Transcription Factors in Regulation of Abiotic Stress Responses in Cotton

**DOI:** 10.3390/life12091410

**Published:** 2022-09-09

**Authors:** Xiaoqiang Guo, Abid Ullah, Dorota Siuta, Bożena Kukfisz, Shehzad Iqbal

**Affiliations:** 1 College of Life Science and Technology, Longdong University, Qingyang 745000, China; 2 Department of Botany, Post Graduate College Dargai, Malakand 23060, Khyber Pakhtunkhwa, Pakistan; 3 Faculty of Process and Environmental Engineering, Lodz University of Technology, Wolczanska Str. 213, 90-924 Lodz, Poland; 4 Faculty of Security Engineering and Civil Protection, The Main School of Fire Service, 01-629 Warsaw, Poland; 5 College of Plant Sciences and Technology, Huazhong Agricultural University, Wuhan 430070, China

**Keywords:** abiotic stresses, cotton, hormones, signaling pathway, WRKY

## Abstract

Environmental factors are the major constraints in sustainable agriculture. WRKY proteins are a large family of transcription factors (TFs) that regulate various developmental processes and stress responses in plants, including cotton. On the basis of *Gossypium raimondii* genome sequencing, WRKY TFs have been identified in cotton and characterized for their functions in abiotic stress responses. WRKY members of cotton play a significant role in the regulation of abiotic stresses, i.e., drought, salt, and extreme temperatures. These TFs either activate or repress various signaling pathways such as abscisic acid, jasmonic acid, salicylic acid, mitogen-activated protein kinases (MAPK), and the scavenging of reactive oxygen species. WRKY-associated genes in cotton have been genetically engineered in *Arabidopsis*, *Nicotiana*, and *Gossypium* successfully, which subsequently enhanced tolerance in corresponding plants against abiotic stresses. Although a few review reports are available for WRKY TFs, there is no critical report available on the WRKY TFs of cotton. Hereby, the role of cotton WRKY TFs in environmental stress responses is studied to enhance the understanding of abiotic stress response and further improve in cotton plants.

## 1. Introduction

As an industrial crop, cotton is cultivated almost all over the world due to its crucial role in the economy of a country [1]. Cotton is generally divided into two types, i.e., wild and cultivated cotton. The cultivated cotton species are *Gossypium hirsutum* (*G*. *Hirsutum*), *G*. *herbaceum*, *G. arboreum*, and *G. barbadense*. Among the cultivated species, *G*. *hirsutum*, sometimes also called “American, Mexican or upland cotton”, is the most cultivated cotton species globally. Despite cultivated cotton species, there are about 46 wild types of cotton species that majorly belong to Australia and Mexico [2]. The importance of cotton fiber as a product is due to its massive use in the textile industry [3,4]. Regarding world cotton production, 80% comes from India, China, the United States of America (USA), Pakistan, Brazil, Turkey, and Uzbekistan. Cotton production is crucial to the economy of several developing countries such as Pakistan, India, and China [5,6]. However, global warming created several types of abiotic stresses, which limit cotton production worldwide.

The harsh environmental conditions, including drought, salinity, extreme temperatures, and high concentration of heavy metals, result in low crop yield and consequently reduce the economy of a country [7,8]. Although cotton is slightly resistant (glycophytic) to environmental stresses compared with rice, wheat, and maize; however, extreme environmental conditions are still affecting cotton production and fiber quality [9]. Due to the immobile nature of plants, they tackle the abiotic factors in the same environment by inducing different biological pathways and the production of stress hormones [10]. Adaptations to these environmental stresses are critical for the life cycle of cotton plants and also for their successive generations. Therefore, numerous adaptations have been developed by cotton plants over the long course of evolution to perceive, transduce, and respond to environmental stimuli by several morph-physiological, cellular, and molecular processes [11,12]. In response to environmental stresses, extensive molecular reprogramming starts in cotton plants at both the transcriptional and post-transcriptional levels. The principal gene regulators, i.e., transcriptional factors (TFs), trigger several stress-responsive genes to mitigate the effect of abiotic stresses in cotton [13,14,15]. These transcription factors include MYB, WRKY, ERF, NAC, and bZIP, which are involved in stress responses and development [9]. WRKY TFs are among the major regulators in cotton, which need to be studied for further improvements in cotton. Hereby, the aim of the present study is to highlight the role of WRKY TFs in cotton against abiotic stresses. In addition, it also explains several molecular signaling pathways associated with WRKY TFs in cotton under abiotic stresses.

## 2. WRKY Transcription Factors

Among the TFs, numerous WRKY TFs have been evaluated in plants for their crucial role in the regulation of stress responses. In addition to stress responses, their role in senescence and development has also been reported [11,13]. The WRKY family is considered one of the largest families of TFs in plants, including cotton. The first member of WRKY transcription factors was studied in sweet potato. Subsequently, several WRKY members have also been identified in other plants, including 74 members in *Arabidopsis*, 109 in rice, 197 in soybean, and 71 in pepper [16,17]. *Arabidopsis* is considered a model plant, and its WRKY proteins are well classified due to their small genome size. WRKY proteins are classified according to the number of WRKY domains and the differences present in the Zinc finger motif. Overall, the members of WRKY proteins are classified into three major groups, i.e., Group I, II, and III, based on the number and diversity of WRKY domains. Group I proteins contain two WRKY domains, while the rest of the two groups contain one in each [18]. On the other hand, Group II and III proteins are differentiated from each other due to the structural differences of the zinc fingers motif. In this way, Group II proteins have a C_2_H_2_ zinc finger motif, while Group III proteins possess a C_2_HXC zinc finger motif [19,20,21]. Moreover, Group II proteins can be further classified into five subgroups (IIa-IIe) based on the phylogenetic analysis of WRKY. Apart from the WRKY domain and zincfinger motif, most members of WRKY transcription factors have nuclear localization signals, a serine/threonine-rich region, leucine zippers, kinase domains, glutamine-rich region, proline-rich region, and other structures. These various structures confer different transcriptional regulatory functions on WRKY TFs [22].

The name WRKY was assigned to these transcription factors after a conserved region of 60 amino acids called the WRKY domain. The domain is characterized by a highly conserved heptapeptide motif (WRKYGQK) at the N-terminal and a zinc finger-like motif at the C-terminal [23,24]. In order to respond to both internal (development) and external stimuli (stresses), members of the WRKY proteins bind to the W-box (TGACC (A/T)) in the promoter region of its target genes and regulate the expression of downstream genes responsible for the development and/or stress [19,25]. This triggering is usually auto-regulated by the WRKY proteins themselves or by another WRKY transcription factor [11]. During normal conditions, the WRKY genes regulate numerous important biological functions related to the developmental processes in cotton and other plants. For example, a comparative transcriptomic study of cotton species during somatic embryogenesis revealed that 4.8% of WRKY TF encoding genes were detected in somatic embryogenesis [26]. Similarly, *GhWRKY15* is involved in the regulation of root and stem development [17]. In addition, WRKY TFs regulate several physiological processes associated with stress response in cotton either by activating or inhibiting the transcription of physiological processes.

## 3. Cotton WRKY Transcription Factors

The sequencing of the *Gossypiumraimondii* genome is provided the opportunity to conduct genome-wide identification of WRKY genes in cotton. Like other plants, i.e., *A. thaliana*, *Triticum aestivum*, *Oryza sativa*, and *Cicer arietinum* [27,28,29,30], genome-wide identification of cotton WRKY genes has also been assessed [31,32,33]. Both the whole genome sequence scaffolds of two drafts of the D_5_ genome [1,31,34] and express sequence tags (ESTs) from four cotton species, Cai et al. [35] detected 120 candidate WRKY genes. These WRKY genes were based on the sequence information from Paterson et al. [33,35]. Of these TFs, 103 homologous WRKY genes were also found based on the sequence data of Wang et al. [1]. In addition, 3668 ESTs, including 70, 148, 519, and 2935 ESTs from *G*. *arboreum*, *G*. *barbadense*, *G*. *raimondii*, and *G*. *hirsutum,* respectively, were found to match these WRKY members with at least one EST hit. When the cotton WRKY genes were compared with *Arabidopsis* sequences present in the online database of TAIR (http://www.arabidopsis.org/), 105 WRKY homologs were also found in *Arabidopsis* [35]. These candidate WRKY genes were unevenly distributed on 13 chromosomes in *G*. *raimondii* from Paterson et al. [34]. Based on the phylogenetic analysis, WRKY members were divided into three major groups, Group I contained 20 members, Group II had 88, and Group III contained 12 members. Group II genes were classified further into five subgroups, groups IIa, b, c, d, and e, which contained 7, 16, 37, 15, and 13 members, respectively [34].

In a genome-wide identification of the WRKY gene family in *G*. *raimondii*, a total of 116 WRKY genes were found from the complete genome sequence. Members of the WRKY family have distributed unevenly on the chromosomes of *G*. *raimondii*, chromosome 7 and 5 contained the largest (16.04%) and fewest (2.83%) number of GrWRKY genes, respectively. When the GrWRKY protein domain structures were compared with AtWRKY proteins, variation in the WRKY domain structure was observed [31]. In another study, the authors of the reference [32] identified 109 and 112 WRKY genes in *G*. *arborium* and *G*. *raimondii*, respectively. According to the physical mapping, WRKY genes in *G*. *arborium* were not present on the same chromosome of *G*. *raimondii*, which revealed that there is a great chromosomal rearrangement in the diploid cotton genome. These studies revealed that there are more than 100 WRKY genes in the *G*. *arborium* or *G*. *raimondii* genome. In addition to general identification of the WRKY gene family, drought, salt, heat, cold, and alkalinity responsive members of the WRKY gene family have also been found [31,32,35,36], which shows its crucial role in abiotic stresses. These TFs enhance the stress-induced gene responsiveness and hence the overall stress tolerance. The expression pattern of a large number of GhWRKY genes was high during leaf senescence and fiber development which shows its prominent role in leaf senescence and fiber development of diploid cotton [32,35,37]. Similarly, WRKY genes *GhWRKY27* and *GhWRKY42* have been reported to induce leaf senescence and anther development in transgenic plants [38,39,40]. The higher expression levels of WRKY transcription factors in different tissues such as root, stem, leaf, petal, and anther reveal their role in the respective tissue [35].

Numerous cotton WRKY genes have been characterized in model plants for their crucial role in modulating stress responses. In a case, overexpression of *GhWRKY15* increased resistance against the infection of viruses and fungi in tobacco [17], while overexpressing *GhWRKY34* and *GhWRKY41* enhanced salt tolerance in *Arabidopsis* and drought tolerance in tobacco, respectively [41,42].

## 4. Functions of WRKY TFs in Cotton against Abiotic Stresses

The effects of environmental stresses and response mechanisms of cotton are discussed below. It is important to understand the response mechanism of WRKY TFs signaling pathways in manipulating the cotton genome against abiotic stresses.

### 4.1. Drought and Heat Stresses

According to the United States Department of Agriculture (USDA), cotton production is expected to reduce by drought stress in the USA and other countries [9]. Similarly, in Pakistan, during the last five years, cotton production declined by 23.7% to an average of 7.42 M bales against the average production of 9.72 M bales from the previous 5 years. In addition to other reasons, Harsh weather is responsible for this decline in cotton production [43,44]. In general, drought conditions restrict plant growth and yield by affecting seed germination, plant height, leaf area index, canopy, fiber quality, and root development [45]. Specifically, it decreases the photosynthetic rate, stomata conduction, transpiration rate, carboxylation efficiency, and water potential of cotton leaves significantly during drought stress [46]. Recently, Ibrahim et al. [47] reported the negative effects of drought stress on cotton plants. According to their study, 31.1% lint yield and 44.4% fresh plant weight of *Gossypium hirsutum* (Zhongmian 41) were reduced under drought stress. In addition, the chlorophyll content and photosynthetic rate were also recorded lower under drought compared with control plants. However, abscisic acid (ABA), indole acetic acid (IAA), superoxide dismutase (SOD), H_2_O_2_, callose, and proline contents were reported to be higher. The effects of drought stress on cotton and its coping strategies have been extensively reviewed in our previous report [9,46]. Plants have devolved numerous morphological, cellular, and molecular adaptations to cope with drought stress. In the case of biochemical adaptations, cotton WRKY TFs are the key regulators in reducing the effects of drought and heat stresses and consequently lead to various morpho-physiological changes crucial for drought and heat tolerance in cotton.

WRKY transcription factors in cotton have been reported widely for their prominent role in the regulation of drought stress [33,39,48,49]. During an investigation, 34 IId WRKY genes were identified in the *G*. *hirsutum* genome. Among these, 10 genes were distinctly expressed under drought and salt stresses. The highly expressed gene, Gh_A11G1801, was silenced by Virus-Induced Gene Silencing (VIGS) technology in cotton plants. The VIGS resulted in cotton seedlings showing enhanced sensitivity to drought stress. In addition, these plants had higher malondialdehyde (MDA) content and lower catalase (CAT) content [33]. Similarly, the expression of group III WRKY genes in cotton was assessed under abiotic stresses. Expression patterns of *GhWRKY7*, *50*, *59*, *60*, and *102* were significantly upregulated under ABA, mannitol, and salt treatments. It reveals that these genes may regulate drought and/or salt stress-responsive pathways, i.e., the ABA signaling pathway in cotton plants. In addition, *GhWRKY7* and *GhWRKY7102* genes were markedly expressed in roots under high concentration (200 mmol L^−1^) of mannitol and NaCl showing their crucial role in root improvement [31]. Moreover, the *GhWRKY1-like* transcription factor was identified in *G. hirsutum* as a drought tolerance regulator. The overexpression of *GhWRKY1-like* in Arabidopsis improved drought tolerance by manipulating ABA synthesis and interaction with several cis-elements [50]. An extensive root system is often counted as favorable for drought tolerance in cotton and other plants. A transcriptomic study of cotton roots under drought stress revealed that *GhWRKY75* is involved in root development [51].

### 4.2. Salt Stress

Salinity is a global problem that affects approximately 20% of irrigated land and reduces crop yields remarkably [52]. As a glycophyte, cotton is tolerant to mild salt stress; however, high salt concentration affects cotton plants in various ways. It causes oxidative stress and ion toxicity and affects nutrient uptake, increases water deficiency, alters metabolic processes, membrane disorganization, and genotoxicity, and reduces cell division and expansion. Consequently, these factors affect cotton growth, development, productivity, and fiber quality [53]. Cotton plants respond to salinity stress by various morpho-physiological and biochemical changes, where several pathways work together at the transcriptional and post-transcriptional levels. At the transcriptional level, WRKY transcription factors are the key mediators in the salt tolerance of cotton.

With the release of the cotton genome sequence, genome-wide analysis of WRKY family genes has been carried out in *G. arboreum, G. raimondii*, and *G. aridum*. Numerous studies have revealed the importance of specific WRKYs in the transcriptional regulation of salt-related genes in cotton [54]. In a transcriptomic analysis of wild-type-salt-tolerant cotton species, 109 GarWRKY genes were identified [55]. Overexpression of a cotton WRKY gene, *GhWRKY25* enhanced salt tolerance in *Nicotiana benthamiana* [56]. Similarly, overexpressing *GhWRKY39-1 Nicotiana benthamiana* plants showed increased salt and oxidative stress tolerance. In addition, overexpression of *GhWRKY39-1* increased the transcriptional level of antioxidant enzyme-associated genes [57]. Moreover, the overexpression of another cotton WRKY gene, *GhWRKY6-like,* markedly enhanced salt tolerance in *Arabidopsis*. On the other hand, the silencing of the *GhWRKY6-like* gene in cotton through VIGS technology increased the sensitivity of cotton plants to drought and salt stresses [58].

### 4.3. Cold Stress

Cold stress is among the major abiotic stresses that limit cotton growth, productivity, and fiber quality [59,60,61]. Plants have devolved sophisticated mechanisms involving altered physiological and biochemical processes to cope with cold stress. The coping strategies that plants develop to tolerate harsh conditions are diverse among plants. These strategies often start changes to protect plants in the first instance, followed by cold acclimation, enhancing plant survival under cold stress [62]. Most of these processes are regulated by TFs that trigger the expression of stress-responsive genes. WRKY transcription factors are among those TFs which mediate cold tolerance in cotton plants.

The expression of WRKY TFs during cold treatment in cotton has been widely reported, which exhibited that WRKY TFs regulate cold-stress response. In a transcriptomic analysis of cotton, 10 WRKY TFs were differentially expressed (upregulated) under cold stress [63]. In a previous study, the expression of *GhWRKY41* was significantly induced by cold (4 °C), heat (37 °C), salt, and drought stresses [41]. Similarly, the *GhWRKY15* expression level was also increased after the treatment of the cold. However, the overexpressing *GhWRKY15* and *GhWRKY41* tobacco lines were only checked for drought, salt, and other stresses [17].

The response of cotton plants to low temperatures is not only limited to the transcription network. As a principal stress hormone, ABA promotes phospholipid metabolism and generates second messengers in cold stress [64]. In a study, cold stress slightly enhanced endogenous ABA levels in plants [65]. While the role of WRKY TFs in the ABA pathway has been discussed in Section 5.2, it reveals that WRKY TFs indirectly regulate responses to cold stress.

### 4.4. Other Abiotic Stresses

Stresses are varied from place to place and time to time; similarly, plants cope with each stress in their own correspondence. In addition to drought, heat, salt, and cold stresses, other abiotic stresses such as waterlog, alkalinity, nutrient deficiency, wounding, and heavy metals stresses have also been reported for cotton. These stresses have also been found to be regulated by WRKY TFs [22,66]. Members of the WRKY gene family were induced in response to waterlog stress. For example, microarray data were used and investigated 50 GhWRKY gene expression patterns in roots and leaves under waterlog stress. As a result, the expression level of several genes was found higher in the root than the in the leaves, which suggests that most of the GhWRKY genes responding to waterlog stress are present in cotton roots. In addition, numerous GhWRKY genes were also induced by pH stress in cotton. The expression level of GhWRKY genes in waterlog and pH stresses suggest the participation of WRKY genes in regulating these stresses [31].

Phosphorus is one of the essential plant nutrients for normal growth and development; however, plants uptake phosphorus in the form of Phosphate [67]. Cotton WRKY gene, *GbWRKY1* regulated Phosphate deficiency in cotton. Overexpressing *GbWRKY1 Arabidopsis* plants reduced Phosphorus deficiency symptoms, accumulated high levels of total phosphorus, increased lateral root development, and Phosphatase activity. These results speculated the positive role of *GbWRKY1* in Phosphate starvation and its involvement in the modulation of Phosphate homeostasis and participation in Phosphate allocation and remobilization [68].

Wounding stress is also counted as one of the abiotic stresses caused by snow, rain, strong wind, herbivores, and insect attack. Wounding tissues are attractive and easy sites for pathogen (bacteria and virus) infection [69]. It activates both local and systematic response mechanisms in plants. The systematic response mechanism includes transcriptional and post-transcriptional changes [70]. In order to validate the role of WRKY TFs in wounding, a cotton WRKY gene, the *GhWRKY15* expression level was markedly high after 2 h of wounding stress [17]. In addition, the transcript level of the cotton WRKY gene, *GhWRKY40*, was increased in cotton upon wounding, while its overexpression enhanced the wounding tolerance in *Nicotiana benthamiana* [71].

Heavy metals are one of the environmental stresses affecting both plants and animals negatively [72]. Upon heavy metal (Cd) stress, several transcription factors, including WRKY, were upregulated in rice [73]. For cotton, although it has been studied successfully in the phytoremediation of heavy metals [74], there is no such report available on the WRKY TF’s involvement in the regulation of heavy metals stress response in cotton. There may have functions of cotton WRKY TFs in the regulation of heavy metal stress or phytoremediation of heavy metals. Thus, it is suggested to study WRKY TFs in cotton under heavy metals stress and phytoremediation.

## 5. Signaling Pathways Associated with WRKY TFs

**Figure 1:**
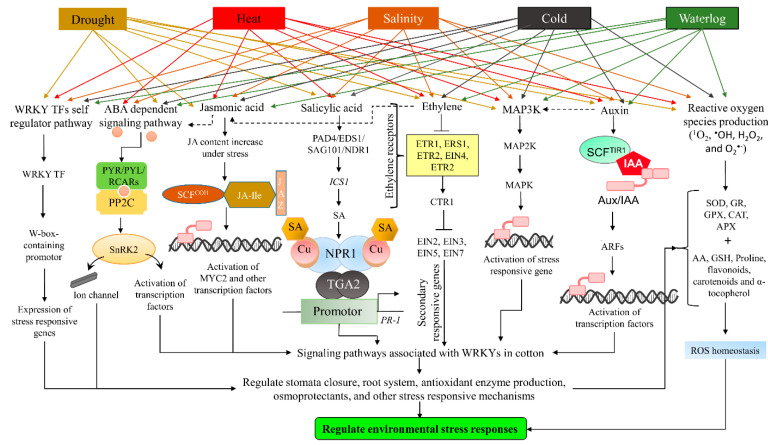
Association of WRKY TFs with stress-responsive signaling pathways during abiotic stresses in cotton.

Cotton has evolved several strategies to cope with abiotic and biotic stresses because they cannot escape from stresses. Numerous phytohormones, Reactive oxygen species (ROS) scavenging, and kinases signaling pathways play a crucial role in signals transmission of abiotic stresses. Phytohormones, ABA, Jasmonate (JA), Auxin (IAA), Salicylic acid (SA), Ethylene (ET), Brassinosteroids (BR), Gibberellin (GA), and Cytokinin (CK) play a key role in regulating cotton plant response against pathogens and abiotic stresses [7,46]. Especially, ABA regulates plant responses against abiotic stresses [15]. These signaling pathways are presented in Figure 1, where several pathways respond to abiotic stresses in a coordinated form.

### 5.1. Self-Regulatory Pathway

WRKY TFs are involved in stress responses by auto-regulation (self-regulation) or cross-regulation. In auto-regulation, WRKY protein binds to a W-box-containing promotor and auto-regulates the expression of stress-related genes. Apart from self-regulation, in cross-regulation, the expression of stress-related genes is regulated by another WRKY TF [75]. The evidence is available for the model plant, i.e., *Arabidopsis*, where AtWRKY18, 40, and 60 interacted with themselves and with each other under stress conditions [11,22]. In addition, group III WRKY proteins, AtWRKY30, 53, 54, and 70, were also interacting with themselves and with each other [76]. Similarly, GhWRKY91 is directly bound to the W-box promotor of GhWRKY17 and cross-regulated GhWRKY17 expression in cotton under drought stress. GhWRKY17 is involved in ABA signaling and ROS production [77]. It revealed that cotton has a cross-regulatory pathway of WRKY under drought stress. The self/cross-regulatory WRKY signaling pathway is shown in the schematic diagram of overall signaling pathways in cotton under stress conditions (Figure 1).

### 5.2. Abscisic Acid

Abscisic acid is a major stress-responsive hormone that plays a crucial role in stress signaling pathways. When plants are exposed to different environmental stresses such as extreme temperatures, drought, and salinity, plant growth is modulated by coordination between several plant hormones, proteins, and regulatory factors [78]. ABA plays an essential role in inducing various responses, such as the closing of the stomatal aperture and the expression of stress-responsive genes under abiotic stresses. Abscisic acid responses to stress conditions are divided into slow and rapid responses [79]. The slow response includes the expression of target genes, while a rapid response is mediated by ion channels. Abscisic acid signaling cascade consists of three steps regulatory process, including receptors, protein kinases, and targets, i.e., transcription factors and ion channels [80]. The perception of ABA through ABA receptors (membrane-bound or soluble) initiated the ABA-mediated signaling cascade under stress conditions. The major component of ABA perception and signaling consists of soluble cytoplasmic PYrabactin Resistance (PYR)/PYrabactin Resistance such as (PYL)/Regulatory Component of ABA Receptors (RCAR) proteins. Protein Phosphatase 2C (PP2Cs) is the negative regulator in the ABA signaling pathway, i.e., it inhibits the SNF1-related kinases (SnRK2). Under stress conditions, ABA rapidly accumulates and binds to the ABA receptors (PYR/PYL/RCARs), which in turn bind and inactivate PP2C and lead to the auto-activation of SnRK2 [81,82,83]. The activated SnRK2 further phosphorylates the downstream targets in the form of TFs and ion channels. The ABA signaling pathway in response to environmental stresses has been presented schematically in Figure 1. Recently, several studies revealed that WRKY TFs are positive regulators of ABA-induced stomatal closure and hence abiotic stress responses, i.e., drought, heat, salt, etc. Further, the role of WRKY TFs in the ABA signaling pathway has been reviewed by the authors of the reference [75] in detail.

WRKY TFs in cotton are key elements in the ABA-mediated signaling network under environmental stresses. Numerous cotton WRKY genes have been testified to be induced by ABA treatment, and the same genes enhanced/reduced abiotic stress tolerance in cotton. In a case study, the overexpression of the cotton WRKY gene, *GhWRKY41,* improved drought and salt tolerance in *Nicotiana benthamiana* by an ABA-dependent signaling pathway. It has been reported widely that ABA regulates stomatal movement. Hereby, stomatal aperture and conductivity were remarkably reduced by ABA treatment. In addition, *GhWRKY41* is highly expressed in stomata. Moreover, the genes associated with ABA signaling were also upregulated during drought or salt stress. Among these, the *SnRK2* gene was also induced significantly by drought or salt treatment, while SnRK2 is directly involved in the ABA signaling pathway [41]. In contrast, *GhWRKY17* increased plant sensitivity to drought stress by reducing the ABA content and expression level of ABA-associated genes. In addition, the accumulation of ROS and the production of antioxidant enzymes were also enhanced in overexpressing *GhWRKY17* plants [48]. These studies revealed the essential role of ABA in cotton and its association with WRKY genes during abiotic stresses.

### 5.3. Jasmonic Acid, Ethylene, and Salicylic Acid

Jasmonic acid (JA) and its active derivatives, jasmonates, serve as important signaling molecules in the regulation of stress responses and play an important role in mediating the expression of defense-associated genes [84]. In addition, JA is also involved in plant growth and development, such as root growth, tendril coiling, fruit ripening, and viable pollen production [85]. On the other hand, SA is another phytohormone that plays an essential role in plant growth, development, and stress response mechanisms [86]. Similarly, ethylene is a naturally occurring gaseous hormone with multiple actions, including growth, fruit ripening, senescence, flowering, seed germination, and response to both biotic and environmental stresses [46,87]. The JA, SA, and ethylene signaling pathways during stress conditions are presented in Figure 1. WRKY TFs have a crucial role in the regulation of these phytohormones under environmental stresses.

WRKY TFs in cotton are also involved in regulating jasmonic acid, ethylene, and salicylic acid signaling pathways [17,88]. Overexpressing *GarWRKY5* increased salt tolerance in transgenic *Arabidopsis* by regulating ROS scavenging, jasmonic acid, and salicylic acid pathways [55]. The overexpressing *GhWRKY40* enhanced wounding tolerance in *Nicotiana benthamiana*. The transcript level of *GhWRKY40* was increased by the stress hormones methyl jasmonate, salicylic acid, and ethylene [71]. The expression pattern of several GhWRKY genes (*GhWRKY5*, *GhWRKY7*, *GhWRKY27*, *GhWRKY31*, *GhWRKY50*, *GhWRKY56*, *GhWRKY59*, *GhWRKY60*, and *GhWRKY102*) were upregulated by one or more treatments of SA, JA, Ethylene, ABA, mannitol, and NaCl [31]. In another case study, *GhWRKY15* was significantly induced by the application of JA and SA [17].

### 5.4. Scavenging of ROS

Reactive oxygen species consist of singlet oxygen (^1^O_2_), hydrogen peroxide (H_2_O_2_), superoxide anion (O_2_^−^), and hydroxyl radical (OH) [89]. Production of ROS enhances under abiotic and biotic stresses beyond the threshold level and causes ROS-related injuries in plants [90]. When the concentration of ROS in a cell reaches beyond the threshold level, it causes oxidative damage to DNA, RNA, proteins, and membranes. Eventually, it may cause program cell death if the oxidation did not control on time [9]. However, plants evolved a complicated scavenging system to control the excess amount of ROS in cells. The plant scavenging system against ROS consists of enzymatic [e.g., SOD, catalase (CAT), guaiacol peroxidase, glutathione reductase, and ascorbate peroxidase] and non-enzymatic mechanisms [e.g., ascorbic acid, glutathione (GSH), proline, flavonoids, carotenoids, and α-tocopherol] [91,92,93]. Like other plants, cotton has also developed a complicated scavenging system to cope with abiotic stresses and relieve the effects of oxidative stress. While WRKY TFs have been studied for their prominent role in mediating ROS scavenging pathways in cotton, the evaluation of a WRKY TF gene, *GhWRKY6-like,* showed an elevated response to oxidative stress. Overexpression of *GhWRKY6-like* reduced the H_2_O_2_ and MDA content in *Arabidopsis* under osmotic stress [58]. Overproduction of ROS in the cell increased the MDA level [94] and is used as a marker for the destructive effects of ROS under stress conditions [92]. In addition, SOD and POD activities were also recorded higher in overexpressing *GhWRKY6-like* transgenic lines under salt and osmotic stresses. Proline played an essential role in oxidative stress responses and was also accumulated higher in overexpressing lines than wild-type (WT) plants under salt and osmotic stresses. They suggested that *GhWRKY6-like* promoted the expression of marker genes associated with the ABA signaling pathway and other stress-responsive genes, thereby enhancing drought and salt tolerance. The enhanced tolerance might be due to the activation of the ABA signaling pathway and improved scavenging system for ROS [58]. The increased antioxidant enzyme activities under salt and drought conditions reflect the reduced MDA level in overexpressing plants suggesting that *GhWRKY6-like* is involved in the scavenging of ROS. In contrast, another WRKY gene, *GhWRKY17,* reduced salt and oxidative stress tolerance in *Nicotiana benthamiana*. Under salt and drought stresses, the overexpressing plants had higher H_2_O_2_ and O_2_^−^ content compared with WT plants, indicating that ROS levels were increased by the ectopic expression of *GhWRKY17* in tobacco plants. On the other hand, antioxidant enzyme activities in overexpressing plants subjected to drought stress exhibited a decreased level of SOD, POD, APX, and CAT content. In addition, proline content was also recorded to be lower in transgenic lines than in WT plants [48]. The reduced antioxidant enzyme activities under salt and drought conditions reflect oxidative damage in overexpressing plants. These results revealed that *GhWRKY17* is involved in the ROS signaling pathway negatively. These contrasting studies reveal that WRKY transcription factors regulate the ROS signaling pathway in both positive and negative manner in plants under environmental stresses. Thus, we suggest determining the role of WRKY genes before up- or downregulation in a plant and whether it positively or negatively regulates the ROS signaling pathway. Upregulate the expression of WRKY genes that positively regulate the ROS scavenging signaling pathway and downregulate the expression if it is negatively involved in regulating the ROS signaling pathway.

### 5.5. Kinases (MAPK)

Mitogen-activated protein kinase (MAPK) cascade is one of the principal pathways mediating plant responses to environmental stresses. The MAPK cascade is involved in transferring extracellular signals to the nucleus for appropriate cellular response [95]. This cascade is minimally consisting of three kinases, a MAPKKK (MAP3K), a MAPKK (MAP2K), and a MAPK, which activate each other in a consecutive manner via phosphorylation (Figure 1) [96]. These MAPKs play a significant role in cellular signaling by transferring information from sensors to responders. Mitogen-activated protein kinase cascades are involved in plant responses to water deficit, extreme temperatures, salinity, wounding, and pathogens [97,98]. WRKY TFs in cotton have been studied in the regulation of MAPK activation.

In a genome-wide identification of the MAPKKK gene family in cotton, the authors of the reference [99] identified 157 GhMAPKKKs. Various cis-elements were identified in the promotor regions of GhMAP3Ks related to stress responses. The transcription levels of maximum genes were significantly altered under abiotic stresses. In addition, the expression of some GhMP3Ks genes, i.e., *GhMEKK1012*, *24*, *31*, *36*, *38*, *40*, *45*, *GhRAF2*, *3*, *4*, *7*, *8*, *21*, *49*, and *GhRAF78* were induced under abiotic stresses in cotton. Silencing of *GhMEKK12* and *GhRAF4* through VIGS technology increased drought sensitivity in cotton seedlings. Drought-related physiological parameters, i.e., relative water content, proline, SOD, and POD contents, were reduced in the gene-silenced seedlings, while the stomatal aperture and MDA contents were enhanced. This study revealed the importance of protein kinases in cotton under abiotic stresses. An earlier study revealed that the cotton WRKY TF gene, *GhWRKY59* played a significant role in the ABA-independent GhMAPK cascade. A GhMAPK cascade consisting of GhMAPKKK15 (MAP3K)-GhMKK4 (MAP2K)-GhMPK6 (MAPK) was identified in cotton by Li et al. [100]. GhWRKY59 regulates MAPK activation and GhMAPKKK expression through feedback. GhWRKY59 binds to the GhDREB2 promoter and regulates the expression of downstream drought-responsible genes. Moreover, it regulates GhMAP3K15 expression positively by establishing a feedback loop. Overexpression of *GhWRKY59* in *Arabidopsis* increased drought tolerance in transgenic plants. A novel phosphorylation loop (GhMAP3K15-GhMKK4-GhMPK6-GhWRKY59) has been found in cotton that regulates the GhDREB2-mediated and ABA-independent drought response, which shows the WRKY-associated MAPK cascade in cotton [100].

The current advancement in the field of plant signaling under stress conditions has revealed that a single factor does not trigger the overall response mechanism in cotton plants. Mostly, these pathways activate each other and work in the form of an integrative signaling mechanism [101]. Under single or multiple stresses, the coordinated response mechanism of cotton in the form of stress signaling pathways associated with WRKY, either directly or indirectly, has been elaborated in the schematic diagram (Figure 1). The tolerance mechanism of cotton to several environmental stresses still needs to be understood due to the complex nature of their response.

## 6. Cotton WRKY TFs Genetically Engineered Plants

Genetically engineered plants could be a way to mitigate the effects of abiotic stresses. Generally, genetic modification in crop plants comes through the introduction of a beneficial foreign gene or silencing of the expression of an endogenous gene [102]. In the last few decades, genetic manipulation has been started in several crop plants to develop stress-resistant varieties [11]. Several crops have been genetically modified successfully with increased crop yield, stress-resistant, and improved food quality [103,104,105]. Although concerns persist in humans about the use of genetically modified crops, 526 transgenic events have been approved to date in more than 30 crops for cultivation in various countries of the world. Among them, *Zea maiys* L. (maize) accounts for the highest number of events, i.e., 238, followed by cotton, i.e., 67, and then others [105,106]. Cotton has also been genetically modified to enhance tolerance against abiotic stresses by overexpressing stress-related genes and/or silencing interested genes. Of these, cotton WRKY genes have also been overexpressed and/or silenced in other plants, mostly in *Arabidopsis* and tobacco. Thus far, all the genetically modified cotton WRKY genes have been enlisted in Table 1. Previous research work revealed that WRKY TFs regulate several mechanisms related to biotic and abiotic stresses. It includes root development, stomatal aperture regulation, phytohormone signaling pathways, the generation of antioxidants and osmoprotectant substances, accumulation of metabolites, and stress-associated gene expression [11,22,66,107]. For instance, overexpression of cotton WRKY gene, *GarWRKY5,* enhanced salt tolerance in *Arabidopsis*. Overexpressing lines accumulated higher levels of antioxidant enzymes, i.e., SOD and POD. In addition, transgenic lines had longer roots than wild type. These characteristics in overexpressing transgenic lines improved salt tolerance, while silencing of *GarWRKY5* in cotton increased plant sensitivity to salt stress [55]. In contrast, the ectopic expression of a GhWRKY gene, *GhWRKY17,* enhanced salt and drought sensitivity in *Nicotiana benthamiana*. The overexpression of *GhWRKY17* reduced root length, seed germination, stomatal closure, chlorophyll content, and expression pattern of ABA-signaling-pathway-associated genes. In addition, increased water loss rate, electrolyte leakage, and accumulation of O_2_^−^, H_2_O_2_, and MDA contents were examined in overexpressing tobacco which led to enhanced salt and drought sensitivity [48].

Most of the cotton WRKY genes were characterized in *Arabidopsis* and tobacco (Table 1), while a few WRKY genes have also been studied in cotton through virus-induced gene silencing technology. However, there is no report available on the overexpression of WRKY genes in cotton, although it has been overexpressed in other crops. For example, overexpressing *TaWRKY2*, a WRKY transcription factor, significantly enhanced grain yield and drought tolerance in transgenic wheat [108]. On the other side, no one tested the yield parameters, which is the most important part for the farmers and industry (Table 1). Till now, we did not find any report on cotton WRKY genes overexpressing in cotton, although several WRKY family genes have been overexpressed in Arabidopsis and tobacco. Thus, it is strongly suggested to select and overexpress the WRKY genes (as summarized in Table 1) in cotton. In addition, it would be highly appreciated if two or more genes regulating different parameters (stress-tolerant, increased yield, high fiber quality, etc.) would be engineered in the cotton.

**Table 1:** Cotton WRKY genes regulated abiotic stresses.

WRKY Gene (Sub Group)	Stress	Cellular Localization	Expression in Plant	Traits Regulated	Reference
*GbWRKY1* (I)	Reduced Phosphorus starvation, and tolerance to drought and salt stresses	Nucleus	Overexpressed in Arabidopsis and cotton	Overexpressing lines reduced the accumulation of anthocyanin and enhanced the activity of phosphatase, MDA content, ion leakage and root inhibition	[68,109]
*GhWRKY3* (I)	Expressed under drought, salt and low temperature	Nucleus	NA	NA	[110]
*GarWRKY5* (III)	Enhanced salt tolerance	NA	Silenced in cotton and overexpressed in Arabidopsis	Overexpressing lines exhibited higher activities of SOD, POD and enhanced root length	[55]
*GhWRKY6*	Regulated drought and salt stresses	NA	Silenced in cotton and overexpressed in Arabidopsis	Overexpressing plants showed shorter root length, larger stomatal aperture, increased H_2_O_2_ and MDA level, reduced proline accumulation and participated in ABA signaling pathway	[111]
*GhWRKY6-like*	Improved salt and drought tolerance	Nucleus	Overexpressed in Arabidopsis and silenced in cotton	In overexpressing lines, MDA and H_2_O_2_ content were reduced and proline, SOD and POD contents were increased. Expression pattern of ABA signaling pathway genes was also reported higher in overexpressing lines.	[58]
*GhWRKY15* (IId)	Increased resistant against wounding Viral and fungal infection	Nucleus	Overexpressed in Tobacco	Increased POD, APX and expression of stress related genes	[12]
*GhWRKY17* (IId)	Enhanced drought and salt sensitivity	Nucleus	Overexpressed in *Nicotiana benthamiana*	Reduced root length, seed germination, stomatal closure, chlorophyll content and expression pattern of marker genes involved in ABA signaling pathway Increased water loss rate, electrolyte leakage and accumulation of O_2_^−^, H_2_O_2_, MDA content	[48]
*GhWRKY25* (I)	Enhanced salt tolerance but reduced drought tolerance and plant defense against fungal pathogen	Nucleus	Overexpressed in *Nicotiana benthamiana*	Increased MDA, O_2_^−^, and H_2_O_2_ content Decreased SOD, POD and CAT activities, inhibited root length under drought stress	[56]
*GhWRKY27a* (III)	Reduced drought tolerance	Nucleus	Overexpressed in *Nicotiana benthamiana* and silenced in cotton	Overexpressing lines exhibited short roots, closed stomata, high content O_2_^−^, and H_2_O_2_, and low level of drought related genes expression	[49]
*GhWRKY33* (III)	Reduced drought tolerance	Nucleus	Overexpressed in Arabidopsis	Inhibited seed germination, early seedling growth and root length, reduced sensitivity to ABA	[24]
*GhWRKY34* (III)	Enhanced salt tolerance	Nucleus	Overexpressed in Arabidopsis	Seed germination, root length, chlorophyll content and expression pattern of stress related genes was higher	[42]
*GhWRKY39* (IId)	Enhanced resistance to pathogen infection and salinity	Nucleus	Overexpressed in *Nicotiana benthamiana*	Reduced hydrogen peroxide accumulation and increased the level of APX, CAT, GST and SOD	[112]
*GhWRKY39-1* (IId)	Enhanced resistance to pathogen infection and salinity	Nucleus	Overexpressed in *Nicotiana benthamiana*	Enhanced root length and expression pattern of SOD, GST, APX, and CAT. Decreased H_2_O_2_ content	[57]
*GhWRKY40* (IIa)	Enhanced tolerance against wounding	Nucleus	Overexpressed in *Nicotiana benthamiana*	Decreased level of O_2_^−^ and H_2_O_2_ Transcript level of JA and SA associated genes in overexpressing plants was decreased	[71]
*GhWRKY41* (III)	Enhanced drought and salinity tolerance	Nucleus	Overexpressed in *Nicotiana benthamiana*	Reduced MDA, H_2_O_2_ content and ABA dependent stomatal opening. Accumulated increased level of SOD, POD and CAT	[41]
*GhWRKY42* (IId)	Induced drought and salinity stress	Nucleus	Overexpressed in Arabidopsis	Increased senescence	[33]
*GhWRKY68* (IIc)	Reduced drought and salt tolerance	Nucleus	Overexpressed in *Nicotiana benthamiana*	Stomatal opening, O_2_^−^, H_2_O_2_, and MDA content were increased. Reduced total chlorophyll, CAT, SOD and POD content, root length, expression pattern of ABA-dependent pathway genes	[113]
*GhWRKY91* (IIe)	Enhanced drought tolerance and delayed senescence	NA	Overexpressed in Arabidopsis	Expression pattern of marker genes involved in drought tolerance was significantly higher.	[77]

Footnote: ABA = Abscisic acid, APX = Ascrobate peroxidase, CAT = Catalase, GST = Glutathione peroxidase, MDA = Malondialdehyde, NA = Not applicable, POD = Peroxidase, SOD = Superoxide dismutase.

## 7. Concluding Remarks, Complications, and Recommendations

Cotton is an important industrial crop, and its production has been hampered badly due to various factors in most of the world. Consequently, reduced the economic growth of several developing countries such as Pakistan, India, China, Brazil, Turkey, etc. [9]. In this regard, research is on the way to improving tolerance in cotton against abiotic stresses. Transcription factors are the major regulators of biological processes involved in abiotic stresses [114]. Among the transcription factors, WRKYs are the largest family of transcription factors regulating several stress-related pathways, including ABA, JA, SA, MAPK, and the scavenging of ROS. Understanding stress-responsive signaling pathways and the consequent manipulation of these could be a gateway for the resistant cotton variety. WRKY transcription factors and their related genes have been expressed in other plants (mostly in Arabidopsis and tobacco), showing significant responses against abiotic stresses. However, the role of those genes/TF may not show the same results in cotton due to its tetra genomic (AD genome) nature. On the other hand, producing transgenic cotton plants require a long time to obtain homozygous transgenic seeds, which disheartens scientists if the resulting transgenic cotton does not produce the same results shown in model plants. Still, various transgenic cotton plants have been produced after a long struggle by scholars. The transgenic cotton plants showed better resistance than existing varieties. Even if successful genetically modified cotton plants are produced, research should still continue to improve and combat future challenges. The available reports on cotton WRKY transcription factors reveal that these TFs regulate abiotic stresses positively and/or negatively.
